# Nitrogen Fertilizer Application and Optimized Planting Density Enhance Rice Yield by Improving the Panicle Type Index and Increasing the Filling Rate of Inferior Grains

**DOI:** 10.3390/plants14111690

**Published:** 2025-05-31

**Authors:** Yanlong Gong, Yue Lei, Zhongni Wang, Hai Xu, Xiaoyi Cheng, Wenfu Chen

**Affiliations:** 1 Guizhou Rice Research Institute, Guizhou Academy of Agricultural Sciences, Guiyang 550006, China; gongyanlong1208@163.com (Y.G.); leiyue0917@163.com (Y.L.); zhuanliwang1@163.com (Z.W.); 2 Rice Research Institute, Shenyang Agricultural University, Shenyang 110866, China

**Keywords:** rice, PTI, nitrogen fertilizer, plant density, yield, grain filling characteristics

## Abstract

This study aimed to investigate the regulatory effects of nitrogen (N) application rate and plant density on panicle type index (PTI), yield, grain filling characteristics, and their correlations. The low-PTI rice variety DP128 (PTI = 0.15) was cultivated under field conditions at four N supply levels (0 (N_0_), 140 (N_140_), 200 (N_200_), and 260 (N_260_) kg∙ha^–1^), and two plant densities (166,755 and 333,495 plants∙ha^−1^). Results showed that N application rate, planting density, and their interactions significantly influenced yield, PTI, grain number in middle/lower secondary branches, and total grain number in lower secondary branches of rice DP128. Parameters trends were consistent over two years. Under N_200_D_10_, the total grain number in lower secondary branches was minimized, while other indices were maximized. Further analysis indicates that under high PTI conditions, the maximum grain-filling rate (*G_max_*), mean grain-filling rate (*G_mean_*), sucrose content, ABA/ETH ratio, and starch content in inferior grains (IGs) were all significantly elevated. Correlation analysis indicated PTI was positively correlated with yield, grain number in middle/lower secondary branches, IGs−*G_max_*, and IGs−*G_mean_* and negatively correlated with the total grain number in the lower secondary branches. In summary, increasing PTI can be achieved by optimizing the distribution of secondary branch grains along the panicle axis, decreasing the number of grains on the lower secondary branches, mitigating the competition for filling materials among inferior grains, improving grain-filling capacity and, ultimately, increasing rice yield.

## 1. Introduction

Rice (*Oryza sativa* L.), one of the world’s staple food crops, plays an indispensable role in ensuring global food security, and enhancing its yield holds paramount significance for sustainable agricultural development [1,2,3,4]. With the acceleration of urbanization and the continuous reduction of cultivated land area, the demand for rice production is increasing [5,6,7,8]. It is projected that global rice production will double by 2050 in order to satisfy the demands of an increasing population [9]. Therefore, enhancing rice yield per unit area and fully exploiting the potential for increased production is crucial to addressing the food challenge. The yield potential of rice is mainly determined by the grain storage capacity (the grain number per panicle) and the degree of filling (grain setting rate and grain weight). Many breeders and cultivators believe that expanding grain storage capacity (i.e., cultivating large-panicle varieties) is one of the main ways to enhance rice yield per unit area. Based on this concept, the International Rice Research Institute has successively bred large-panicle rice varieties, including Chinese interspecific hybrid rice and super hybrid rice [2,10,11]. However, these large-panicle rice varieties have not fully achieved the expected high-yield targets in actual production. The main reason is that the inferior grains in the lower part of the panicle have poor filling and low grain quality, which seriously limits the full realization of their high-yield potential [3,12,13].

There are differences between superior grains (SGs) and IGs in the spikelets on the rice panicle. Generally, SGs are the spikelets on the upper primary stem of the rice panicle, which have the characteristics of early flowering, fast filling rate, and high filling degree. The IGs are the spikelets grown on the secondary branches at the base of the rice panicle, which is characterized by late flowering, low filling rate, and poor filling [14]. Since the flowering and filling processes of SGs and IGs are not synchronized, the grain weight and fruit-setting rate of IGs are significantly lower than those of SGs. The difference in grain weight and fullness between SGs and IGs is particularly evident in large-panicle rice [15,16]. Therefore, improving the fullness of the grains in the lower part of the large-panicle rice is critical to further increase rice yield. Chen et al. [17] suggested that the low grain weight of large-panicle rice may be due to insufficient supply of assimilates. When the supply of photosynthetic assimilates is limited, they are preferentially allocated to the SGs, resulting in a decrease in the weight of the IGs. However, other studies have shown that during the grain filling stage of rice, the soluble sugar content, sucrose concentration, and soluble carbohydrate concentration in the IGs were higher than those in the SGs [18,19,20]. In addition, Kato [21] found that after removing some SGs in the upper and middle parts of the rice panicles, the fullness of the grains on the secondary branches at the base of the rice panicle did not improve significantly. These results suggest that insufficient supply of assimilates is not the main factor limiting the filling of IGs. Li et al. [22] found that by adjusting the source–sink ratio, the grains on the secondary branches at the base of the panicle could not reach the grain weight level of SGs. Although the vascular bundle area of the inferior branches was larger, the grain number carried per unit vascular bundle area was smaller, resulting in poor fullness of the IGs. Therefore, the distribution of secondary branch grains on the panicle axis should be optimized to concentrate them in the middle and upper parts of the panicle axis. This approach minimizes the number of IGs in the lower part of the panicle, effectively enhancing the overall yield and quality of rice.

The different distribution trends of grains from secondary branches on the panicle axis are important factors affecting grain sets. Although the grain number on secondary branches is greatly affected by environmental factors, its distribution characteristics on the panicle axis are mainly determined by genetic factors [23]. In order to more intuitively and accurately reflect the distribution characteristics of grains on secondary branches on the panicle axis, Xu et al. [24] proposed the concept of panicle type index (PTI), which is defined as the ratio of the panicle axis node of the primary branch with the largest grain number on the secondary branch to the total number of primary branches (0 < PTI ≤ 1). When PTI > 0.5, secondary stem seeds are predominantly concentrated in the upper portion of the panicle axis; when PTI = 0.5, they are located in the middle portion; when PTI < 0.5, they are distributed in the lower portion. Xu et al. [25] discovered that, among rice varieties in Liaoning Province, high PTI varieties exhibited significantly higher yields compared to low PTI varieties. They further suggested that enhancing PTI is an effective strategy for balancing yield and quality through the coordination of panicle traits. However, there are currently few research reports on the relationship between rice yield and PTI. Therefore, it is of great significance to deeply explore the impact of PTI on yield and its related mechanisms. In this study, a rice variety with a low PTI (DP128, PTI = 0.15) was used as the test material. Four N supply levels and two planting densities were established to investigate (1) the effects of N fertilizer and planting density on PTI and its relationship with yield, (2) the correlation between PTI and the filling characteristics of inferior grains, and (3) the physiological mechanisms underlying the role of PTI in enhancing rice yield. The findings could provide a theoretical foundation and innovative insights for improving rice yield.

## 2. Results

### 2.1. Yield and Yield Components

The N application rate, planting density, and nitrogen–density interaction significantly affect the yield of the low PTI variety DP128, and the yield variation trend was consistent in the two years (Table 1). With the increase in the N application rate, the yield showed a trend of first increasing and then decreasing. Compared with the N_0_ treatment, the average yield of DP128 in two years increased by 2.499 t∙ha^–1^ following N application, representing a 28.16% increase. In addition, when the planting density increased from D_20_ to D_10_, the average yield of DP128 increased by 2.04 t∙ha^–1^ over two years, representing an increase of 5.99%. Under the N_200_D_10_ treatment, the yield of DP128 reached its maximum, with an average yield of 10.15 t∙ha^–1^ over two years.

Except for planting density having no significant effect on thousand-grain weight (TKW), the N application rate and planting density significantly influenced the grain number per panicle (GPP), the panicle number per hectare (P), the filled grain rate (F), and the TKW of the DP128 rice variety (Table 1). However, the interaction between N and density significantly influenced the GPP and the P, while it did not have a significant impact on the F or TKW. This trend remained consistent throughout both years. With the increase in the N application rate, the P of DP128 showed an upward trend. When the planting density increased from D_20_ to D_10_, the average increase in the P reached 23.18% in two years. With the increase in N application rate, the GPP showed a trend of first increasing and then decreasing. Compared with the N_0_ treatment, the average increases in the GPP in the N_140_, N_200_, and N_260_ treatments were 3.56%, 18.42%, and 14.84%, respectively. When the planting density increased from D_20_ to D_10_, the average decrease in the GPP in the two years was 7.31%. The F of DP128 decreased with the increase in N application, but when the planting density increased from D_20_ to D_10_, the two-year average F increased by 7.56%. Overall, the effects of N application rate and planting density on TKW did not reach a significant level.

### 2.2. PTI

**Figure 1:**
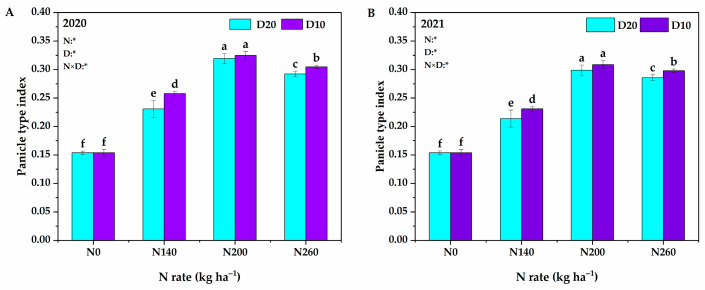
Effects of N application rate and plant density on PTI in low-PTI rice cultivars in 2020 and 2021 (A,B). PTI denotes the panicle type index. N_0_, N_140_, N_200_, and N_260_ represent N applications of 0, 140, 200, and 260 kg∙ha*^−^*^1^, respectively; D_20_ and D_10_ indicate planting density of 166,755 and 333,495 plants∙ha*^−^*^1^, respectively. *signify that the N application rate, planting density, and their interactions had significant effects on the PTI at the 0.05 significance levels. Vertical bars indicate the standard deviation. Different lowercase letters above the bars represent significant differences at the 0.05 significance level.

N application rate, planting density, and their interaction significantly affected the PTI of DP128 (Figure 1). With the increase in N application, the PTI of DP128 showed a trend of increasing and then decreasing, and the variation trend was consistent during the two years. Specifically, the two-year average significantly increased from 0.154 to 0.313 (averaged across both planting densities) when the N application rate was raised from 0 kg·ha^−1^ to 200 kg·ha^−1^. Conversely, the two-year average slightly decreased from 0.313 to 0.295 (also averaged across both planting densities) when the N application rate was further increased from 200 kg·ha^−1^ to 260 kg·ha^−1^. In addition, when the planting density increased from D_20_ to D_10_, the two-year average increased from 0.243 to 0.255 (the average under four N fertilizer levels). Comprehensive analysis showed that the PTI attained its maximum value under N_200_D_10_ treatment.

### 2.3. Panicle Traits

**Figure 2:**
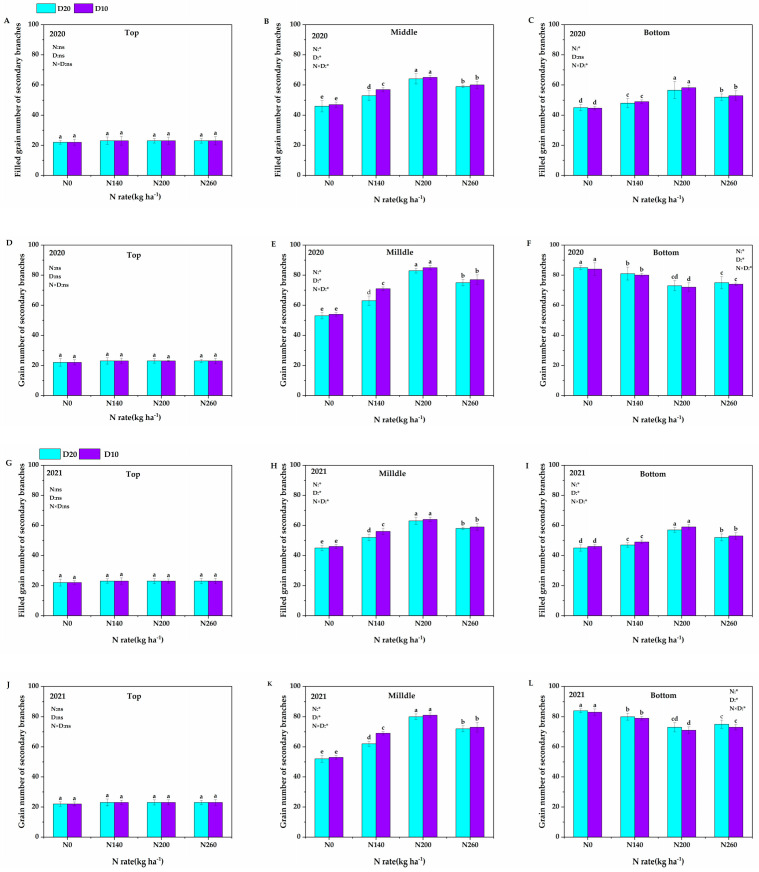
Effects of N application rate and plant density on grain number of secondary branches in low-PTI rice cultivars. N_0_, N_140_, N_200_, and N_260_ represent N applications of 0, 140, 200, and 260 kg∙ha^−1^, respectively; D_20_ and D_10_ indicate planting densities of 166,755 and 333,495 plants∙ha^−1^, respectively. (**A**,**G**) Filled grain number of secondary branches of the top panicle; (**B**,**H**) filled grain number of secondary branches of the middle panicle; (**C**,**I**) filled grain number of secondary branches of the bottom panicle; (**D**,**J**) grain number of secondary branches of the top panicle; (**E**,**K**) grain number of secondary branches of the middle panicle; and (**F**,**L**) grain number of secondary branches of the bottom panicle. * signify that the N application rate, planting density, and their interactions had significant effects on the PTI at the 0.05 significance levels. Vertical bars indicate the standard deviation. Different lowercase letters above the bars represent significant differences at the 0.05 significance level. The symbol “ns” denotes nonsignificant effects.

As shown in Figure 2, the N application rate, planting density, and their interaction significantly influenced the number of filled grains and the total grain number in the middle and lower secondary branches of the panicle. However, these factors did not significantly affect the number of filled grains and total grain number in the upper secondary branches of the panicle. Additionally, the dynamic variation trend was consistent across the two years. With the increase in the N application rate, the number of filled grains and total grains in the middle of the panicle and the number of filled grains in the lower part of the panicle first increased and then decreased. In contrast, the total number of secondary stems in the lower panicle first decreased and then increased. Compared with the N_0_ treatment, the total grain number on the secondary branches below the panicle decreased by 4.19%, 13.47%, and 11.08% in the two-year average under the N_140_, N_200_, and N_260_ treatments, respectively. In addition, when the planting density increased from D_20_ to D_10_, an increasing trend was observed in the number of filled grains and the total grain number on the secondary branches in the middle of the panicle, as well as the number of filled grains on the secondary branches in the lower part of the panicle. Specifically, over two years, the average increase rates were 3.08%, 4.09%, and 2.28%, respectively. Conversely, the total grain number of the secondary branches in the lower part of the panicle showed a downward trend, with an average decrease of 2.236% over the same period.

### 2.4. Grain Distribution of Secondary Branches on the Panicle Axis

**Figure 3:**
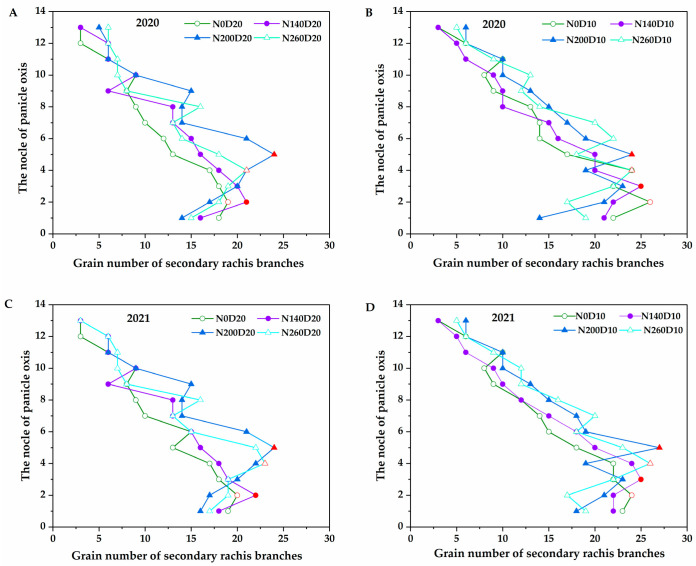
Effects of N application rate and plant density on the grain distribution of secondary branches on the panicle axis in low-PTI rice cultivars. N_0_, N_140_, N_200_, and N_260_ represent N applications of 0, 140, 200, and 260 kg∙ha^−1^, respectively; D_20_ and D_10_ indicate planting densities of 166,755 and 333,495 plants∙ha^−1^, respectively. Red indicates the largest secondary branch grain number of primary branches on the panicle axis. (**A**,**C**) the grain distribution of secondary branches on the panicle axis under D_20_ conditions in 2020 and 2021; (**B**,**D**) the grain distribution of secondary branches on the panicle axis under D_10_ conditions in 2020 and 2021.

As illustrated in Figure 3, with the increasing N application rate, the position of the primary branch containing the highest grain number on the secondary branch of DP128 along the panicle axis first increased and then decreased, showing a consistent trend over the two years. When the N application rate increased from 0 to 200 kg∙ha^–1^, the position of the primary branch with the largest grain number on the secondary branch shifted from the second node of the panicle axis (nodes numbered from bottom to top) to the fifth node. When the N application rate further increased from 200 to 260 kg∙ha^–1^, this position reverted from the fifth node to the fourth. In addition, when the planting density increased from D_20_ to D_10_, the position of the primary branch with the largest grain number on the secondary branch increased from the second node to the third node. Therefore, N fertilizer and planting density primarily influence the changes in PTI by altering the position of the primary branch with the highest grain number on the secondary branch along the panicle axis.

### 2.5. The Correlation of PTI with Yield and the Grain Number of Secondary Branches

**Figure 4:**
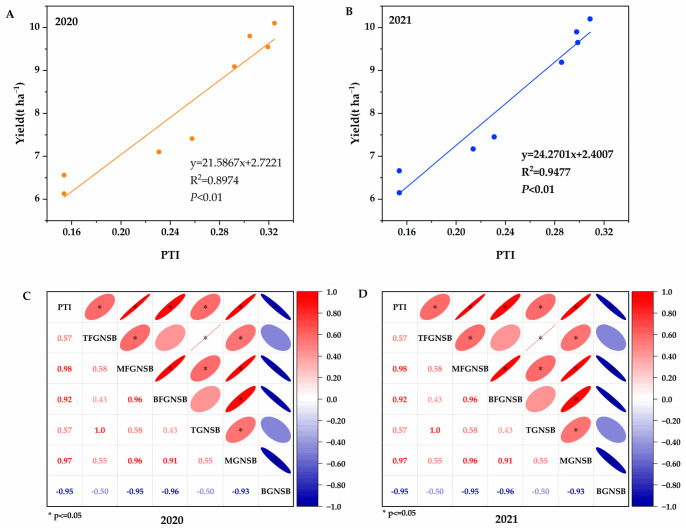
Correlation analysis of yield, grain number of secondary branches, and PTI. PTI denotes panicle type index; TFGNSB denotes the filled grain number of secondary branches of the top panicle; TGNSB denotes the grain number of secondary branches of the top panicle; MFGNSB denotes the filled grain number of secondary branches of the middle panicle; MGNSB denotes the grain number of secondary branches of the middle panicle; BFGNSB denotes the filled grain number of secondary branches of the bottom panicle; and BGNSB denotes the grain number of secondary branches of the bottom panicle. (**A**,**B**) correlation analysis of yield and PTI in 2020 and 2021; (**C**,**D**) correlation analysis of grain number of secondary branches and PTI in 2020 and 2021.

The correlation analysis results show that in 2020 and 2021, the yield exhibited a highly significant positive linear correlation with PTI, with correlation coefficients (R^2^) of 0.8974 and 0.9477, respectively (Figure 4A,B). Furthermore, PTI demonstrated a significant positive correlation with the grain number on secondary branches in the upper, middle, and lower sections of the panicle, as well as the total grain number on secondary branches in the upper and middle sections. Conversely, it showed a significant negative correlation with the total grain number on secondary branches in the lower section of the panicle (Figure 4C,D).

### 2.6. Grain Filling Characteristics of Superior and Inferior Grains

**Figure 5:**
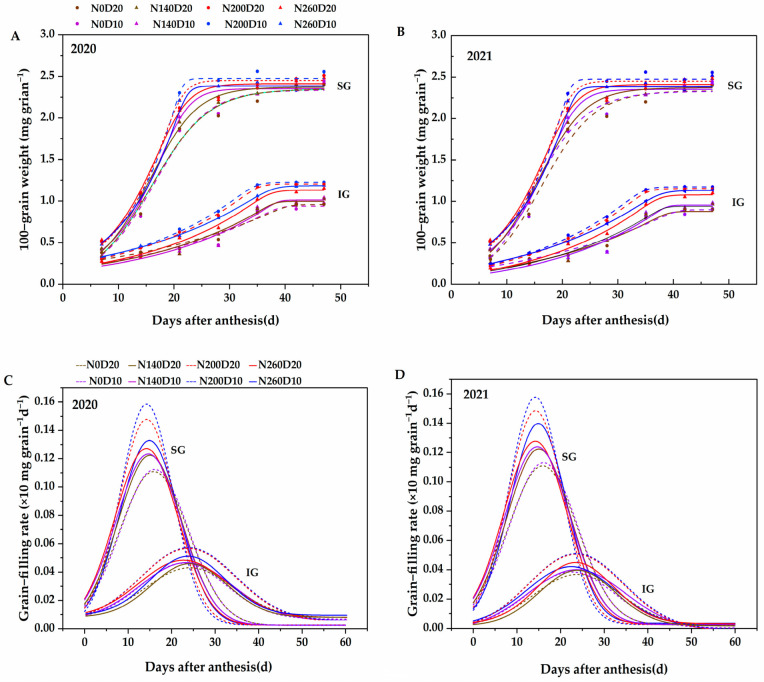
Effects of nitrogen application rate and plant density on grain weight (**A**,**B**) and grain filling rate (**C**,**D**) in low-PTI rice cultivars. N_0_, N_140_, N_200_, and N_260_ represent N applications of 0, 140, 200, and 260 kg∙ha^−1^, respectively; D_20_ and D_10_ indicate planting densities of 166,755 and 333,495 plants∙ha^−1^, respectively. The solid lines represent the conditions of N_0_ and N_200_, while the dotted lines indicate the conditions of N_140_ and N_260_.

With days after flowering as the independent variable and rice grain weight as the dependent variable, the Richards growth equation was employed to fit the weight gain process of SGs and IGs in low-panicle rice (DP128) under varying nitrogen fertilizer and planting density conditions (Figure 5A,B). By calculating its derivative, the grain filling rate curve was further obtained. The determination coefficients (*R*^2^) of the fitting curves for each treatment were all above 0.96, indicating that under varying N fertilizer and density conditions, the filling processes of both SGs and IGs of DP128 rice could be accurately modeled using the Richards equation. This demonstrates the high feasibility of applying the Richards equation in this context.

As depicted in Figure 5C,D, compared with IGs, SGs exhibited a rapid increase in grain weight after flowering, reaching their maximum value approximately 25 to 30 days post-flowering, followed by stabilization. The grain weight of the IGs increased slowly from 0 to 20 days after flowering. After the grain weight of the SGs approached the maximum value, the grain weight of the IGs began to increase rapidly and reached the maximum value 35 to 40 days after flowering. Under the N_200_D_10_ treatment conditions, the grain weight gain curves of the SGs and IGs were both at the top.

The characteristic parameters of SGs and IGs filling of low-panicle rice DP128 under different N fertilizers and density levels were calculated according to the Richards equation. N application rate, planting density, and their interaction had different effects on various parameters of DP128 (Table 2). The maximum grain filling rate (*G_max_*) and mean grain filling rate (*G_mean_*) of SGs were generally higher than those of IGs, indicating that the filling process of SGs was faster and the final grain weight was higher. In contrast, the filling process of IGs was significantly delayed. Their filling rate increased markedly only after the filling rate of the SGs started to decline. Consequently, the time for maximum grain filling rate (*T_max_*) and the duration of active grain filling (*D*) were longer for the IGs than for the SGs.

With the increase in the N application rate, the changing trends of grain filling parameters of SGs and IGs of DP128 are as follows: The grain weight with the maximum grain filling rate (*W_max_*), the *G_max_*, and the *G_mean_* exhibited an overall trend of initially increasing and subsequently decreasing. In contrast, D and *T_max_* demonstrated a pattern of first shortening and then extending. Compared with the N_0_ treatment, the *W_max_*, *G_max_*, and *G_mean_* of the IGs under the N_200_ treatment reached the maximum values, with average increases of 6.11%, 12.58%, and 13.55% in two years, respectively. At the same time, D and *T_max_* were shortened by about 3-4 days. In addition, when the planting density increased from D_20_ to D_10_, the *W_max_*, *G_max_*, and *G_mean_* of the IGs significantly increased, with average increments of 3.81%, 1.11%, and 2.73% over two years, respectively. Meanwhile, D and *T_max_* showed a significant decrease, with an average reduction of approximately 1–2 days across the two years.

### 2.7. The Correlation of Grain Filling Parameters with Yield and PTI

**Figure 6:**
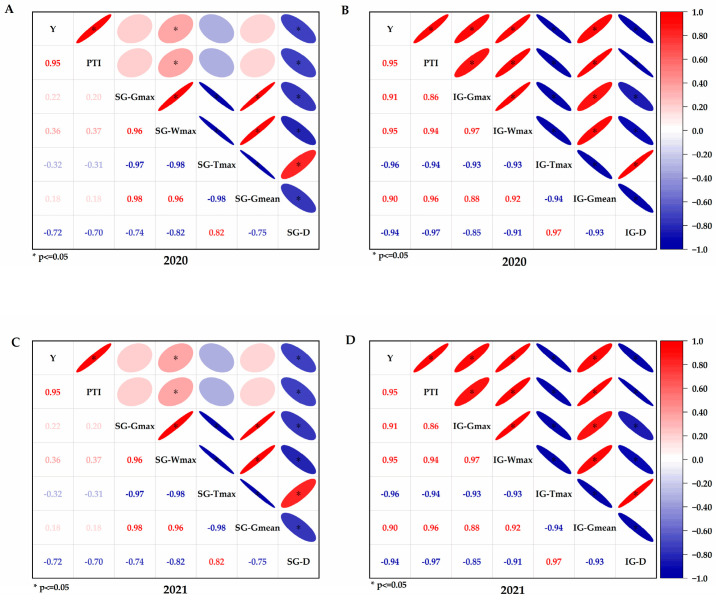
The correlation between grain filling parameters and both yield and PTI. Y, yield; PTI, panicle type index; SG−*G_max_*, maximum grain filling rate of superior grain; SG−*W_max_*, grain weight with the maximum grain filling rate of superior grain; SG−*T_max_*, time for maximum grain filling rate of superior grain; SG-*G_mean_*, mean grain filling rate of superior grain; SG−*D*, active grain filling period of superior grain; IG−*G_max_*, maximum grain filling rate of inferior grain; IG−*W_max_*, grain weight with the maximum grain filling rate of inferior grain; IG−*T_max_*, time for maximum grain filling rate of inferior grain; IG−*G_mean_*, mean grain filling rate of inferior grain; IG−*D*, active grain filling period of inferior grain. (**A**,**C**) correlation between SGs filling parameters and both yield and PTI in 2020 and 2021; (**B**,**D**) correlation between IGs filling parameters and both yield and PTI in 2020 and 2021.

The correlation analysis showed that in 2020 and 2021 (Figure 6), the yield was significantly positively correlated with SG−*W_max_* but significantly negatively correlated with SG−*D_max_*. The yield was significantly positively correlated with IG−*W_max_*, IG−*G_max_*, and IG-*G_mean_* but significantly negatively correlated with IG−*T_max_* and IG−*D_max_*. Similarly, PTI was significantly positively correlated with SG−*W_max_* but significantly negatively correlated with SG−*D_max_*. Meanwhile, PTI was significantly positively correlated with IG−*W_max_*, IG−*G_max_*, and IG−*G_mean_* but significantly negatively correlated with IG−*T_max_* and IG−*D_max_*.

### 2.8. Sucrose Content

**Figure 7:**
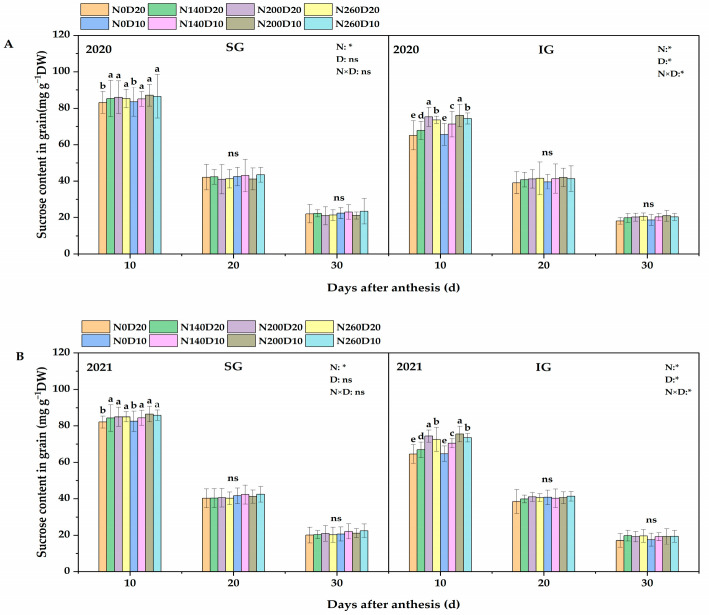
Effects of N application rate and plant density on sucrose content (**A**,**B**) in low−PTI rice cultivars. N_0_, N_140_, N_200_, and N_260_ represent N applications of 0, 140, 200, and 260 kg∙ha^−1^, respectively; D_20_ and D_10_ indicate planting densities of 166,755 and 333,495 plants∙ha^−1^, respectively. * signify that the N application rate, planting density, and their interactions had significant effects on the PTI at the 0.05 significance levels. ns indicate no significance. Vertical bars indicate the standard deviation. Different lowercase letters above the bars represent significant differences at the 0.05 significance level.

At 10 days after flowering, the N application rate, planting density, and the interactions between the two exhibited significant effects on the sucrose content of the IGs of DP128 rice but less significant effects on that of the SGs (Figure 7). Only the N application rate showed a significant effect on the sucrose content of the SGs. The trends observed during the two years were consistent. Specifically, the sucrose content of DP128 IGs showed a trend of first increasing and then decreasing with the increase in the N application rate. Compared with the N_0_ treatment, the two-year average increments in the sucrose content of IGs in the N_140_, N_200_, and N_260_ treatments reached 4.19%, 13.47%, and 11.08%, respectively. Moreover, the sucrose content of IGs showed a significant upward trend as the planting density increased from D_20_ to D_10_, with a two-year average increase of 3.08%. Under the N_200_D_10_ treatment, the sucrose content of DP128 IGs reached the maximum.

### 2.9. ABA/ETH

**Figure 8:**
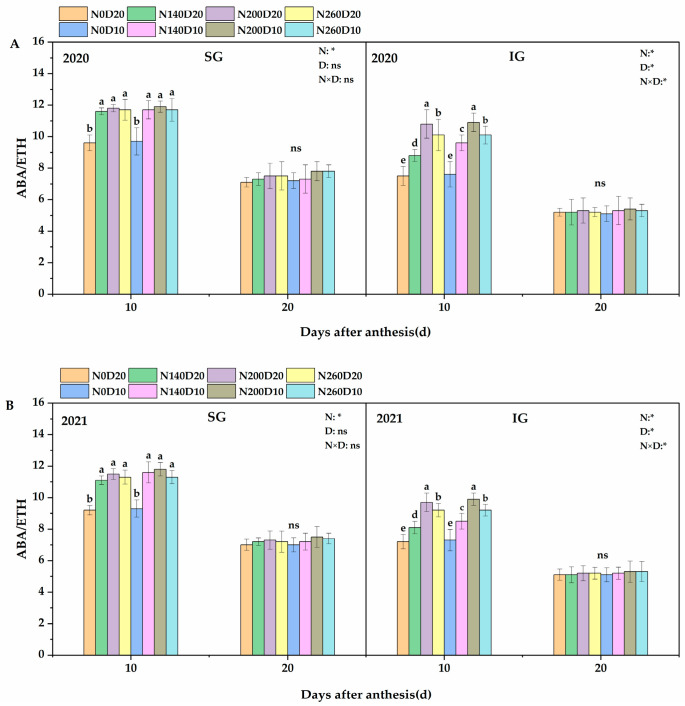
Effects of N application rate and plant density on ABA/ETH (**A**,**B**) in low−PTI rice cultivars. N_0_, N_140_, N_200_, and N_260_ represent N applications of 0, 140, 200, and 260 kg∙ha^−1^, respectively; D_20_ and D_10_ indicate planting densities of 166,755 and 333,495 plants∙ha^−1^, respectively. * signify that the N application rate, planting density, and their interactions had significant effects on the PTI at the 0.05 significance levels. ns indicate no significance. Vertical bars indicate the standard deviation. Different lowercase letters above the bars represent significant differences at the 0.05 significance level.

Ten days after flowering, the N application rate, planting density, and their interactions exhibited significant effects on the ABA/ETH ratio of the IGs of DP128 rice (Figure 8). Meanwhile, their effects on the ABA/ETH ratio of the SGs were less significant, and only the N application rate showed a significant effect. The trends observed in the two years were consistent. The ABA/ETH ratio of DP128 IGs showed a trend of first increasing and then decreasing with the increase in N application rate. Compared with the N_0_ treatment, the two-year average increments in the ABA/ETH ratio of IGs in the N_140_, N_200_, and N_260_ treatments reached 15.43%, 28.33%, and 23.32%, respectively. In addition, the ABA/ETH ratio of IGs increased significantly as the planting density increased from D_20_ to D_10_, with a two-year average increase of 8.36%. In the N_200_D_10_ treatment, the ABA/ETH ratio of DP128 IGs reached the maximum.

### 2.10. Starch Content

**Figure 9:**
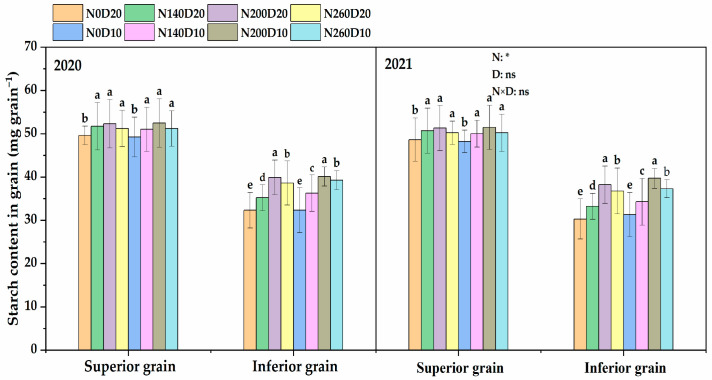
Effects of N application rate and plant density on starch content in superior and inferior grains. N_0_, N_140_, N_200_, and N_260_ represent N applications of 0, 140, 200, and 260 kg∙ha^−1^, respectively; D_20_ D_10_ indicate planting densities of 166,755 and 333,495 plants∙ha^−1^, respectively. Vertical bars indicate the standard deviation. Different lowercase letters above the bars represent significant differences at the 0.05 significance level.

The N application rate, planting density, and their interactions exhibited significant effects on the starch content of the IGs of DP128 rice (Figure 9). Their effects on the starch content of the SGs were less significant, and only the N application rate showed a significant effect. The trends observed in the two years were consistent. With the increase in the N application rate, the starch content of DP128 IGs showed a trend of first increasing and then decreasing. Compared with the N_0_ treatment, the two-year increments of the starch content of IGs in the N_140_, N_200_, and N_260_ treatments increased by 15.43%, 28.33%, and 23.32%, respectively. Additionally, the starch content of IGs increased significantly as the planting density increased from D_20_ to D_10_, with a two-year average increase of 8.36%. In the N_200_D_10_ treatment, the starch content of DP128 IGs reached the maximum.

## 3. Discussion

### 3.1. Effects of Nitrogen Application Rate and Planting Density on the Yield of Low Panicle Type Index Rice Varieties

This study reveals that in 2020 and 2021, as the N application rate increased from 0 kg∙ha^–1^ to 200 kg∙ha^–1^, the yield of DP128 correspondingly increased from 6.345 t∙ha^–1^ and 6.405 t∙ha^–1^ to 9.825 t∙ha^–1^ and 9.925 t∙ha^–1^, respectively. However, upon further increasing the N application rate from 200 kg∙ha^–1^ to 260 kg∙ha^–1^, the yield decreased to 9.445 t∙ha^–1^ and 9.545 t∙ha^–1^ (average values across the two planting densities), respectively. These results align with those reported in previous studies. In addition, numerous studies found that planting density significantly affects the grain number per panicle, and appropriately increasing the planting density can compensate for the spikelet maldevelopment due to nitrogen deficiency to a certain extent, thereby increasing the yield [1,8]. Zhu et al. [26] pointed out that reasonable dense planting can significantly improve the light energy utilization rate and photosynthetically effective radiation interception rate of rice canopy, thereby promoting yield improvement. The significant yield increase in this study as the planting density increases from D_20_ to D_10_ further validates their conclusion.

The synergistic interaction between N fertilizer and planting density plays a crucial regulatory role in the formation of rice yield. This mechanism optimizes the light distribution characteristics of the canopy and enhances the spatio-temporal allocation efficiency of N, thereby establishing a model for the coordinated optimization of resource-efficient utilization and population structure, which significantly increases yield per unit area. Under appropriate planting density conditions, scientifically optimized N fertilizer application can effectively promote tillering and panicle formation, improve the photosynthetic efficiency of the plant population, and achieve dynamic equilibrium in the source-sink relationship. Conversely, combinations of high density with low nitrogen or low density with high N may result in an imbalance in the supply and demand of photosynthetic products, thereby weakening yield potential [1,8]. The results of this study showed that the yield of DP128 rice maximized under the N application rate of 200 kg∙ha^−1^ and the planting density of D_10_, reaching a two-year average yield of 10.15 t∙ha^−1^. In conclusion, the synergistic interaction between N fertilizer and planting density has significant practical significance for increasing rice production.

### 3.2. Relationship Between Yield and Panicle Type Index

Previous research on the yield components of high-yield rice groups showed that “large storage capacity” is an important feature supporting high rice yield [27]. Prior studies have suggested that increasing the number of grains per panicle, particularly the grain number on secondary branches, represents a critical strategy for enhancing storage capacity [28,29]. Nevertheless, in large-panicle rice varieties, nutrient competition often leads to a reduced grain setting rate in the lower portion of the panicle, which constitutes a significant bottleneck for achieving higher yields [30]. This suggests that solely increasing the number of florets may not be adequate to consistently enhance grain yield. It is essential to concurrently improve the efficiency of grain filling. Wu et al. [30] found that optimizing the spatial arrangement of secondary pedicels and bract flowers (e.g., reducing inter-pedicel spacing and enhancing rachis architecture) can improve nutrient allocation efficiency, leading to an increased seed set rate in IGs. Xu et al. [24] studied the PTI differences among the rice varieties in Liaoning Province and found that the yield of high-PTI rice varieties was significantly higher than that of low-PTI varieties, suggesting improving PTI as an effective way to coordinate rice yield and quality.

The results of this study showed that N application rate, planting density, and the interactions between the two significantly affected the PTI of DP128 rice. In the N_200_D_10_ treatment, the PTI of DP128 rice reached the maximum. Correlation analysis showed an extremely significant linear positive correlation between yield and PTI, indicating that increasing rice PTI can effectively promote yield increase. Further comparative analysis of the numbers of filled grains and all grains of the secondary branches in the upper, middle, and lower parts of the panicle showed that increasing PTI significantly increased the number of filled grains of the secondary branches in the middle and lower parts of the panicle but reduced the number of all grains of the secondary branches in the lower part of the panicle. Correlation analysis results showed that PTI was significantly positively correlated with the number of filled grains of the secondary branches in the middle and lower parts of the panicle but significantly negatively correlated with the total number of grains of the secondary branches in the lower part of the panicle. These results indicate that increasing PTI can promote the distribution and concentration of secondary branch grains from the lower part of the axis to the upper and middle parts, thereby optimizing the overall distribution pattern of secondary branch grains on the axis. This distribution optimization can significantly increase the number of filled grains of the secondary branches at the lower part of the panicle and effectively improve the seed setting rate of the secondary branches at the lower part of the panicle, which, in turn, supports further rice yield improvement.

### 3.3. Relationship Between Inferior Grain Filling Characteristics and PTI

The grain filling characteristics are determined by the filling time, the filling rate, or the combined effects of the two, whereas the N fertilizer application rate and planting density are the key regulating factors of grain filling characteristics. Starch is the main component of rice grains, usually accounting for over 70% of their dry weight. Sucrose translocation from the vegetative organs to the grains and starch synthesis within the grains exert important impacts on grain filling characteristics and grain weight [31]. However, IGs exhibit significantly reduced sucrose input compared to SGs, primarily attributed to the weakened connection of vascular bundles or insufficient expression of sucrose transport proteins (e.g., SUTs) [11,14]. The results of this study indicate that under high PTI conditions, the sucrose content in the IGs of rice is significantly higher than that under low PTI conditions. This indicates that raising PTI can shift the distribution and concentration of secondary branch grains to the upper part of the axis, thus effectively reducing the filling material competition among the IGs at the lower part of the panicle. Such an adjustment facilitates greater sucrose accumulation in IGs, providing an adequate substrate for starch synthesis.

The key steps in sucrose transport and starch synthesis are regulated by the hormone signaling network. Studies have demonstrated that a higher ABA/ETH ratio can significantly promote the sucrose translocation to the grains and enhance the starch synthesis efficiency within the grains, thereby increasing the filling rate and TGW [4]. This study revealed that, under high PTI conditions, the ABA/ETH ratio in the IGs of rice was significantly elevated compared to that under low PTI conditions. This indicates that enhancing PTI can optimize the microenvironment, integrate sucrose-hormone signaling, reprogram gene expression, and synergistically suppress ethylene synthesis while enhancing ABA function. Consequently, this improves the grain-filling characteristics of IGs. This mechanism offers a critical theoretical foundation for the breeding of the “ideal plant type” and the regulation of cultivation practices.

In summary, optimizing PTI can significantly accelerate and intensify the filling of IGs, thus improving the filling degree of IGs and supporting rice yield increment.

## 4. Materials and Methods

### 4.1. Experimental Site and Materials

The experiments were conducted at the Shenyang Agricultural University Experimental Base (41°48′ N, 123°34′ E) in Shenhe District, Shenyang City, Liaoning Province, in 2020 and 2021. The rice variety for the experiments was a low-PTI DP128 (PTI = 0.15). The basic soil fertility of the 0 to 20 cm arable layer, which served as the control condition for the experimental variants, included an organic matter content of 26.3 g·kg⁻^1^, total nitrogen of 0.87 g·kg⁻^1^, total phosphorus of 1.1 g·kg⁻^1^, total potassium of 8.93 g·kg⁻^1^, rapidly available potassium of 113.59 mg·kg⁻^1^, rapidly available phosphorus of 61.3 mg·kg⁻^1^, and rapidly available N of 100.3 mg·kg⁻^1^.

### 4.2. Experimental Design

The field experiment was laid out in a split-plot design with 4 N fertilizer levels 0 N kg∙ha^–1^ (N_0_), 140 N kg∙ha^–1^ (N_140_), 200 N kg∙ha^–1^ (N_200_), and 260 N kg∙ha^–1^ (N_260_) as the main plot, and 2 planting densities, 166755(D_10_) and 333,495 (D_20_) plants∙ha*^−^*^1^, as subplots. Each experiment plot was 20 m^2^. Each plot was designed with four N fertilizer treatments and two density treatments, and each treatment was repeated 3 times, resulting in a total of 12 plots. The plots were separated by 0.4 m wide furrows covered with plastic films to ensure isolated drainage and irrigation and prevent water and fertilizer exchanges between plots.

The experiments adopted a thermal-insulated greenhouse substrate dry nursery to cultivate seedlings. Rice was sown on 16 April 2020, and 17 April 2021, transplanted in the 3.5-leaf stage, and harvested between September 22 and 26 each year. One seedling was transplanted per hill. Urea (46% N), potassium chloride (60% K_2_O), and calcium superphosphate (12% P_2_O_5_) served as the N, potassium (K_2_O), and phosphorus (P_2_O_5_) fertilizers applied in each treatment. In the basic fertilization of the experiment, the application rates of K₂O and P₂O₅ were set at 90 kg·ha⁻^1^. Specifically, the base fertilizer consisted of 50% N, 60% K_2_O, and the entire amount of P_2_O_5_. The tiller fertilizer was applied with 25% N and 20% K_2_O, while the panicle fertilizer was applied with the remaining 25% N and 20% K₂O. Each plot was subjected to regular manual weeding and pest and disease control with pesticides, while the other managements followed the local field management methods.

### 4.3. Research Contents and Methods

#### 4.3.1. Panicle Type Index

Before harvesting in the mature stage, the number of panicles of 1 none-edge row with even growth was investigated in each plot. Ten representative medium rice plants were selected from each plot according to the average number of particles to investigate the number of primary branches of all panicles of the 10 plants. After air-drying, 10 panicles were selected for seed testing according to the mode of the number of primary branches. The primary branches were labeled from the bottom to the top of the axis, the grain number of the secondary branches was investigated, and PTI was calculated.

PTI = the position of the primary branch with the highest grain number on its secondary branches/the number of primary branches.

#### 4.3.2. Panicle Traits

At the mature stage, 10 rice plants with uniform growth were selected from the middle of each plot. After air-drying, 10 panicles were selected for seed testing according to the mode of the number of primary branches. Each panicle was divided equally into the upper, middle, and lower parts according to the position of the primary branches. In the case of a remainder (1 or 2 primary branches), it was assigned to the middle part after assigning the quotients to the three parts. The numbers of filled and unfilled grains of the secondary branches in the upper, middle, and lower parts of the panicle were investigated.

#### 4.3.3. Classification of Superior and Inferior Grains

At the initial heading stage, 300 panicles of basically the same size (with around 5 cm of the panicle emerging from the flag leaf sheath) were selected and tagged. From the flowering stage to the mature stage, 15 of the tagged panicles were collected every 7 days. The SGs and IGs were separated, and the unfertilized empty grains were removed. The SGs and IGs were weighed after drying and dehusking. Each panicle was divided equally into the upper, middle, and lower parts according to the position of the primary branches. In the case of a remainder (1 or 2 primary branches), it was assigned to the middle part after assigning the quotients to the three parts. The grains directly born on the primary branches at the top of the panicle (except the second grain at the top) were the SGs, and the grains directly born on the secondary branches of the primary branches at the lower part of the panicle (except the first grain at the top) were the IGs.

#### 4.3.4. Calculation of Rice Filling Parameters

The grain filling process was simulated using the Richards growth equation. The number of days after flowering served as the independent variable t (d), and the grain growth *W* (mg∙grain^−1^) served as the dependent variable following the methodology outlined by Zhu et al. [32].

(1) $$\begin{array}{c} W=\frac{A}{{{(1+Be}^{-kt})}^{\frac{1}{N}}} \end{array}$$

where *A* is the final weight of the grain (mg∙grain^–1^), *k* is the growth rate parameter, and *B* and *N* are the curve setting parameters of the equation. Let the numbers of days where the grain weight reaches 5% and 95% of *A* during the filling process be *t*_1_ and *t*_2_, respectively, and the length of the effective filling stage is defined as *D* = *t*_2_ − *t*_1_.

(2) $$\begin{array}{c} G_{mean}=\frac{1}{D}\int_{t2}^{t1}Gdt \end{array}$$

where *G* is the grain filling rate (mg∙grain^−1^∙d^−1^), *G_mean_* is mean grain-filling rate, and *d* is the differential symbol, which is the first derivative of Equation (1).

(3) $$\begin{array}{c} G=\frac{dW}{dt}=\frac{{AkBe}^{-kt}}{{N(1+Be}^{-kt}{)}^{\frac{N+1}{N}}} \end{array}$$

where *W* is the grain weight (g), *A* is the final grain weight (g), *t* is the time after flowering (d), and *B*, *K*, and *N* are the parameters of the equation.

(4) $$\begin{array}{c} T_{max}\frac{\ln B\;-\ln A}{K} \end{array}$$

where *T_max_* is the time for maximum grain filling rate.

(5) $$\begin{array}{c} D=\frac{A}{\;G_{mean}}=\frac{2\left(N+2\right)}{K} \end{array}$$

*D* is the active growth period.

### 4.4. Determination of Sucrose, Starch, Abscisic Acid, and Ethylene Contents in Rice Grains

According to the experimental method proposed by Liu et al. [33], the sucrose starch, abscisic acid, and ethylene contents were determined.

### 4.5. Statistical Analysis

Data from each treatment were analyzed using one-way analysis of variance (ANOVA) via SPSS 29.0 software (SPSS Institute Inc., Chicago, Unite States). Prior to the analysis, the data were tested for normality using the Kolmogorov–Smirnov test and for homogeneity of variance using the Bartlett–Box test. The results confirmed that the data followed a normal distribution and exhibited homogeneous variance. Subsequently, mean comparisons were performed using the least significant difference (LSD) test at the 0.05 significance level. Graphs were constructed using Origin 2024 software, and the data in the graphs are presented as the mean ± standard deviation.

## 5. Conclusions

Increasing PTI can significantly improve the filling degree of IGs. Specifically, increasing PTI drives the distribution and concentration of secondary branch grains at the lower part of the panicle to the middle and upper parts of the axis, thereby reducing the grain number of secondary branches at the lower part of the panicle and effectively reducing the intensity of filling material competition among the IGs. This optimized distribution can significantly enhance the number of filled grains in the secondary branches at the lower part of the panicle and effectively improve the seed-setting rate of these secondary branches. In addition, the IGs exhibit a high ABA/ETH ratio under a high PTI. This increased ratio helps to promote the sucrose translocation to the grains and enhance the starch synthesis efficiency within the grains. As a result, the filling of IGs is intensified and accelerated, which significantly improves their filling degree and ultimately improves the yield.

## Figures and Tables

**Table 1:** Effects of N application rate and planting density on the grain yield and yield components in rice with low PTI.

	Treatments	Y [t∙ha^–1^]	P [×10 ha^–1^]	GPP	F [%]	TKW [g]
2020	N_0_D_20_	6.13 ± 0.17h	125.5 ± 12.4g	218.4 ± 0.58f	75.6 ± 0.40c	25.13 ± 0.32a
	N_140_D_20_	7.10 ± 0.15f	152.9 ± 22.0f	228.7 ± 3.21e	73.3 ± 1.70d	25.01 ± 0.58a
	N_200_D_20_	9.55 ± 0.14c	165.6 ± 16.3e	270.3 ± 2.31a	63.6 ± 1.40e	25.80 ± 0.43a
	N_260_D_20_	9.09 ± 0.14d	178.6 ± 15.0d	256.0 ± 1.20b	62.7 ± 1.10e	25.39 ± 0.25a
	N_0_D_10_	6.56 ± 0.25g	175.1 ± 16.2d	206.2 ± 2.08g	82.5 ± 1.60a	25.42 ± 0.41a
	N_140_D_10_	7.41 ± 0.03e	198.1 ± 34.6c	209.3 ± 5.51g	79.6 ± 0.90b	25.98 ± 0.48a
	N_200_D_10_	10.1 ± 0.03a	206.4 ± 34.1b	248.0 ± 2.20c	72.2 ± 1.80c	25.74 ± 0.41a
	N_260_D_10_	9.80 ± 0.02b	228.2 ± 32.2a	241.0 ± 2.10d	63.0 ± 1.60e	24.29 ± 0.26a
2021	N_0_D_20_	6.15 ± 0.34h	129.3 ± 15.1g	221.0 ± 3.00f	76.7 ± 1.00c	25.66 ± 0.45a
	N_140_D_20_	7.17 ± 0.06f	156.4 ± 12.1f	235.1 ± 4.04e	74.7 ± 1.10d	25.45 ± 0.89a
2021	N_200_D_20_	9.65 ± 0.13c	169.1 ± 15.3e	276.0 ± 1.73a	65.3 ± 3.20e	25.04 ± 0.70a
	N_260_D_20_	9.19 ± 0.17d	180.6 ± 13.3d	260.0 ± 1.22b	63.0 ± 2.20f	25.04 ± 0.37a
	N_0_D_10_	6.66 ± 0.20g	178.1 ± 16.2d	208.0 ± 4.90g	83.0 ± 2.30a	25.60 ± 0.56a
	N_140_D_10_	7.45 ± 0.17e	200.4 ± 21.2c	212.0 ± 2.00g	80.0 ± 1.00b	25.20 ± 0.26a
	N_200_D_10_	10.2 ± 0.06a	216.1 ± 20.0b	252.0 ± 1.73c	75.0 ± 1.80d	25.01 ± 0.56a
	N_260_D_10_	9.90 ± 0.28b	235.1 ± 22.4a	245.3 ± 1.15d	65.0 ± 0.60e	24.02± 0.44b
ANOVA	N level (N)	**	**	**	**	**
	Density (D)	**	**	**	**	ns
	Yield(Y)	*	**	ns	ns	ns
	N × D	*	**	**	ns	ns
	Y × N	*	**	ns	ns	ns
	Y × D	ns	**	ns	ns	ns
	Y × N × D	ns	ns	ns	ns	ns

Note: Y represents the yield, P denotes the panicle number per hectare, GPP indicates the grain number per panicle, F represents the filled grain rate, and TKW represents the 1000-grain weight. N_0_, N_140_, N_200_, and N_260_ represent N applications of 0, 140, 200, and 260 kg∙ha^−1^, respectively; D_20_ and D_10_ indicate planting density of 166,755 and 333,495 plants∙ha^−1^, respectively. Data are expressed as the mean ± SD of triplicates. Asterisks (*) and (**) signify statistically significant influences of N rate, planting density, and their interactions on yield and yield components at the 0.05 and 0.01 significance levels, respectively. The symbol “ns” denotes nonsignificant effects. Values of the eight treatments marked with different lowercase letters indicate significant differences at the 5% significance level.

**Table 2:** Effects of nitrogen application rate and plant density on grain filling parameters of SGs and IGs.

Year	Treatment	*R^2^*		*G_max_* (mg grain^−1^d^−1^)		*W_max_* (mg grain^−1^)		*T_max_*(d)		*G_mean_* (mg grain^−1^d^−1^)		*D* (d)	
		SG	IG	SG	IG	SG	IG	SG	IG	SG	IG	SG	IG
2020	N_0_D_20_	R^2^ = 0.9692	R^2^ = 0.9907	1.125	0.421	11.52	8.48	15.11	25.26	0.782	0.457	35.11	39.13
	N_140_D_20_	R^2^ = 0.9707	R^2^ = 0.9703	1.148	0.436	12.71	8.78	14.29	23.21	0.787	0.511	35.9	37.36
	N_200_D_20_	R^2^ = 0.9954	R^2^ = 0.9983	1.46	0.53	13.51	10.28	11.01	20.95	0.810	0.564	33.99	35.67
	N_260_D_20_	R^2^ = 0.9913	R^2^ = 0.9685	1.271	0.458	12.3	9.46	12.38	21.73	0.803	0.521	34.47	35.51
	N_0_D_10_	R^2^ = 0.9948	R^2^ = 0.9930	1.127	0.422	11.61	8.26	14.01	24.25	0.792	0.477	35.69	39.06
	N_140_D_10_	R^2^ = 0.9933	R^2^ = 0.9792	1.151	0.442	12.99	8.99	13.33	22.53	0.791	0.528	35.4	36.72
	N_200_D_10_	R^2^ = 0.9917	R^2^ = 0.9987	1.597	0.545	13.78	10.36	11.07	19.03	0.851	0.567	32.11	34.36
	N_260_D_10_	R^2^ = 0.9980	R^2^ = 0.9910	1.302	0.498	13.33	9.95	11.56	20.84	0.806	0.549	33.24	35.87
2021	N_0_D_20_	R^2^ = 0.9945	R^2^ = 0.9993	1.123	0.417	11.49	8.38	15.09	25.28	0.762	0.443	35.55	39.33
	N_140_D_20_	R^2^ = 0.9902	R^2^ = 0.9721	1.145	0.433	12.66	8.65	14.31	23.26	0.765	0.501	35.76	37.45
	N_200_D_20_	R^2^ = 0.9839	R^2^ = 0.9994	1.454	0.511	13.43	10.11	11.11	21.05	0.813	0.551	33.93	35.72
	N_260_D_20_	R^2^ = 0.9957	R^2^ = 0.9885	1.266	0.452	12.21	9.32	12.42	21.76	0.801	0.521	34.55	35.53
	N_0_D_10_	R^2^ = 0.9948	R^2^ = 0.9937	1.124	0.422	11.51	8.22	14.09	24.28	0.772	0.469	35.76	39.26
	N_140_D_10_	R^2^ = 0.9854	R^2^ = 0.9994	1.148	0.438	12.84	8.71	13.39	22.61	0.783	0.511	35.48	36.82
	N_200_D_10_	R^2^ = 0.9953	R^2^ = 0.9913	1.593	0.541	13.69	10.15	11.13	19.11	0.829	0.551	32.23	34.46
	N_260_D_10_	R^2^ = 0.9951	R^2^ = 0.9974	1.301	0.495	13.21	9.65	11.60	20.86	0.786	0.531	33.56	35.82

Note: SG and IG denote superior and inferior grains, respectively; N_0_, N_140_, N_200_, and N_260_ represent N applications of 0, 140, 200, and 260 kg∙ha*^−^*^1^, respectively; D_20_ and D_10_ indicate planting densities of 166,755 and 333,495 plants∙ha*^−^*^1^, respectively. *G_max_*, maximum grain filling rate; *W_max_*, grain weight with the maximum grain filling rate; *T_max_*, time for maximum grain filling rate; *G_mean_*, mean grain filling rate; *D*, active grain filling period.

## Data Availability

The data used to support the findings of this study can be made available by the corresponding author upon request. The data are not publicly available due to the fund information and other papers that have not been published, and these data will not be uploaded.

## References

[1] Liu Y., Liao Y., Liu W. (2021). High nitrogen application rate and planting density reduce wheat grain yield by reducing filling rate of inferior grain in middle spikelets. Crop J..

[2] Dastan S., Ghareyazie B., Silva J.A.T.D. (2020). Selection of ideotype to increase yield potential of *GM* and *non-GM* rice cultivars. Plant Sci..

[3] Liao C., Yan W., Chen Z., Xie G., Deng X., Tang X. (2021). Innovation and development of the third-generation hybrid rice technology. Crop J..

[4] Chen L., Deng Y., Zhu H., Hu Y., Jiang Z., Tang S., Wang S., Ding Y. (2019). The initiation of inferior grain filling is affected by sugar translocation efficiency in large panicle rice. Rice.

[5] Chen W.-f., Xu Z.-j., Tang L. (2017). 20 years’ development of super rice in China—The 20th anniversary of the super rice in China. J. Integr. Agric..

[6] Tian Q., Zheng D., Chen P., Yuan S., Zhen X. (2025). The Effects of reducing nitrogen and increasing density in the Main Crop on yield and *cadmium* accumulation of ratoon rice. Agronomy.

[7] Li H., Geng J., Liu Z., Ao H., Wang Z., Xue Q. (2025). Mulching improves the yield and water use efficiency of millet in northern China: A Meta-Analysis. Agriculture.

[8] Hou W., Khan M.R., Zhang J., Lu J., Ren T., Cong R., Li X. (2019). Nitrogen rate and plant density interaction enhances radiation interception, yield and nitrogen use efficiency of mechanically transplanted rice. Agric. Ecosyst. Environ..

[9] Hu Q., Liu Q., Jiang W., Qiu S., Wei H., Zhang H., Liu G., Xing Z., Hu Y., Guo B. (2021). Effects of mid-stage nitrogen application timing on the morphological structure and physicochemical properties of japonica rice starch. J. Sci. Food Agric..

[10] Zhang Z., Gao S., Chu C. (2020). Improvement of nutrient use efficiency in rice: Current toolbox and future perspectives. Theor. Appl. Genet..

[11] Yang J., Zhang J. (2010). Grain-filling problem in ‘super’ rice. J. Exp. Bot..

[12] Wang Z., Xu Y., Chen T., Zhang H., Yang J., Zhang J. (2015). Abscisic acid and the key enzymes and genes in sucrose-to-starch conversion in rice spikelets in response to soil drying during grain filling. Planta.

[13] Sekhar S., Gharat S.A., Panda B.B., Mohaptra T., Das K., Kariali E., Mohapatra P.K., Shaw B.P. (2015). Identification and characterization of differentially expressed genes in inferior and superior spikelets of rice cultivars with contrasting panicle-compactness and grain-Filling properties. PLoS ONE.

[14] Yang J.C. (2010). Mechanism and regulation in the filling of inferior spikelets of rice. Acta Agron. Sin..

[15] Huang L., Yang D., Li X., Peng S., Wang F. (2019). Coordination of high grain yield and high nitrogen use efficiency through large sink size and high post-heading source capacity in rice. Field Crops Res..

[16] Liu K., Zhang K., Zhang Y., Cui J., Li Z., Huang J., Li S., Zhang J., Deng s., Zhang Y. (2024). Optimizing the total spikelets increased grain yield in rice. Agronomy.

[17] Chen D.W., Zhang G.P., Yao H.G., Wu W., Wang R.Q. (2003). Studies on the grain-filling properties of compact panicle type of rice. Acta Agron. Sin..

[18] Mu X., Chen Q., Chen F., Yuan L., Mi G. (2016). Within-Leaf nitrogen allocation in adaptation to low nitrogen supply in maize during grain-filling stage. Front. Plant Sci..

[19] Shen L.-x., Huang Y.-k., Li T. (2017). Top-grain filling characteristics at an early stage of maize (*Zea mays* L.) with different nitrogen use efficiencies. J. Integr. Agric..

[20] Sun Y., Ma J., Sun Y., Xu H., Yang Z., Liu S., Jia X., Zheng H. (2012). The effects of different water and nitrogen managements on yield and nitrogen use efficiency in hybrid rice of China. Field Crops Res..

[21] Kato T. (2004). Effect of spikelet removal on the grain filling of Akenohoshi, a rice cultivar with numerous spikelets in a panicle. J. Agric. Sci..

[22] Li X.T., Cheng H.T., Wang N., Yu C.M., Qu L.Y., Cao P., Hu N., Liu T., Lyu W.Y. (2013). Critical factors for grain filling of erect panicle type *Japonica* rice cultivars. Agron. J..

[23] Sasahara T., Kodama K., Kambayashi M. (1982). Studies on structure and function on the rice ear. Jpn. J. Crop Sci..

[24] Xu Z.J., Chen W.F., Sun Z.H., Zhang S.L., Liu L.X., Zhou S.Q. (2004). Distribution of rice grain on panicle axis and its relationship with seed setting in Liaoning. Sci. Agric. Sin..

[25] Xu Z.J., Chen W.F., Zhang S.L., Zhang W.Z., Ma D.R., Liu L.X., Zhou S.Q. (2005). Differences of panicle trait index among varieties and its relationship with yield and quality of rice in Liaoning. Sci. Agric. Sin..

[26] Zhu X.C., Tang L., Zhang W.Y., Cao M.Y., Cao W.X., Zhu Y. (2012). Transfer characteristics of canopy photosynthetically active radiation in different rice cultivars different cultural conditions. Sci. Agric. Sin..

[27] Sheehy J.E., Dionora M.J.A., Mitchell P.L. (2001). Spikelet numbers, sink size and potential yield in rice. Field Crops Res..

[28] Dong G.C., Tian H., Zhang B., Li J.Q., Yu X.F., Wang Y.L. (2009). Characteristics of source-sink related parameters in conventional indica rice cultivars with different types of sink potential. Acta Agron. Sin..

[29] Wang F.Y., Huang P.S. (1997). Study on source-sink characteristics and high-yield cultivation strategies of rice population. Sci. Agric. Sin..

[30] Wu W.G., Zhang H.C., Wu G.C., Zhuo C.Q., Qian Y.F., Chen Y., Xu J., Dai Q.G., Xu K. (2007). Preliminary study on super rice population sink characters. Acta Agron. Sin..

[31] Yang W., Li Y., Yin Y., Qin Z., Zheng M., Chen J., Luo Y., Pang D., Jiang W., Li Y. (2017). Involvement of ethylene and polyamines biosynthesis and abdominal phloem tissues characters of wheat caryopsis during grain filling under stress conditions. Sci. Rep..

[32] Zhu Q.S., Cao X.Z., Luo Y.Q. (1988). Gromth analysis on the process of grain filling in rice. Acta Agron. Sin..

[33] Liu Y., Ding Y., Wang Q., Meng D., Wang S. (2011). Effect of nitrogen and 6-benzylaminopurine on rice tiller bud gowth and changes in endogenous hormones and nitrogen. Crop Sci..

